# Diabetes, minor depression and health care utilization and expenditures: a retrospective database study

**DOI:** 10.1186/1478-7547-5-4

**Published:** 2007-04-18

**Authors:** Lori Nichols, Phoebe L Barton, Judith Glazner, Marianne McCollum

**Affiliations:** 1 Department of Preventive Medicine and Biometrics, University of Colorado at Denver and Health Sciences Center, Denver, Colorado, USA; 2 Department of Clinical Pharmacy, University of Colorado at Denver and Health Sciences Center, 4200 East Ninth Ave., Box C-238, Denver, Colorado, 80262 USA

## Abstract

**Background:**

To estimate the prevalence of minor depression among US adults with diabetes, health care resource utilization, and expenditures by people with diabetes with and without minor depression.

**Methods:**

Among adult 2003 Medical Expenditure Panel Survey respondents, diabetes was identified by diagnosis code and self-report. Depression was identified by diagnosis code plus ≥ one antidepressant prescription. Odds of having depression was estimated in people with diabetes and the general population, adjusted for sociodemographic variables (e.g., age, sex, race/ethnicity). Multivariate regressions evaluated factors associated with utilization and log-transformed expenditures for ambulatory care, hospitalizations, emergency visits, and prescriptions.

**Results:**

In 2003, 1932 respondents had diabetes, 435/1932 had diabetes and minor depression. Adults with diabetes were more likely than the general population to have depression (adjusted OR 1.81, 95% CI 1.56, 2.09). People with diabetes with versus without comorbid depression were more likely to be women, have lower incomes and health status, and more diabetes complications (all p < 0.05). In unadjusted analyses, ambulatory care visits were higher for those with versus without depression (17.9 vs. 11.4, p = 0.04), as were prescriptions (60.7 vs. 38.1, p = 0.05). In adjusted analyses, depression was not associated with increased resource use or higher expenditures in any category. Increased number of comorbid conditions was associated with increased resource use in all categories, and increased expenditures for ambulatory care and prescriptions.

**Conclusion:**

People with diabetes are twice as likely to have depression as the general population. Screening for and treatment of depression is warranted, as is additional research into a causal relationship between diabetes and depression.

## Background

Diabetes and depression are costly chronic medical conditions, suffered by millions of Americans every year. The American Diabetes Association estimates that 20.8 million people had diabetes in 2005.[1] In 2002, total expenditures for people with diabetes in the U.S. (including all inpatient care, outpatient care and pharmaceuticals for all health conditions – not only diabetes care) were estimated to be nearly $132 billion.[2] In 2000 the prevalence of depression in the United States was between 5 and 10.3 percent of the population, affecting more than 19 million Americans each year.[3] The economic burden of depression in 2002 was estimated at $83.1 billion.[4,5]

Co-occurrence of these two illnesses is prevalent in the United States. People with diabetes are approximately 2.5 times more likely to be diagnosed with depression than those without diabetes. [6-8] Temporal or causal relationships between the two conditions remain unclear but under study.

Health care expenditures and utilization among individuals with diabetes and comorbid depression warrant additional research for a number of reasons. Depressed patients with a chronic illness are less likely to follow a recommended health or medication regimen.[9] Depressive symptoms are associated with a perception of more impaired physical health and may mimic worsening diabetes symptoms.[9] Poor glucose control is associated with depression and effective treatment of depression may improve diabetes control.[10]

Previous studies evaluating health care expenditures and utilization associated with diabetes and comorbid depression report that in regional or selected populations, comorbid diabetes and depression are associated with more health care utilization and expenditures than diabetes without depression.[9,11,12] The most recent study estimating odds of diagnosed depression in individuals with diabetes and relationships between depression and health care utilization and expenditures in a nationally representative database was performed using 1996 data from the Medical Expenditure Panel Survey (MEPS). We propose to update that analysis, and hypothesize, based on recent drug developments in both diabetes management and treatment of depression, that individuals with both diabetes and minor depression will have higher rates of health care utilization in four categories: ambulatory care, inpatient care, emergency department visits, and prescription medications. While an increase in health care utilization often predicts a proportionate increase in expenditures, this may not hold true due to improved overall health resulting from effective treatment. Therefore, expenditures in all four categories will also be examined for US adults with diabetes with and without minor depression.

## Methods

### Data Source

Study subjects were respondents in the 2003 MEPS database. MEPS is cosponsored by the Agency for Healthcare Research and Quality (AHRQ) and the National Center for Health Statistics and is representative of the US non-institutionalized civilian population.[13] MEPS uses sampling weights reflecting adjustments for survey non-response and population totals from the Current Population Survey.[13] As a result, MEPS weights were applied in all analyses to obtain nationally representative estimates.

### Case Definition for Study Subjects

The study cases were defined as individuals with diabetes with and without comorbid depression, identified using *International Classification of Diseases, Ninth Revision, Clinical Modification *(ICD-9-CM) codes. MEPS uses three digit (less specific) ICD-9-CM codes rather than five digit codes in order to protect respondent confidentiality. ICD-9-CM code 250, Diabetes Mellitus, was used to identify people with diabetes. Diabetes was also identified using patient self-report in answer to survey questions asking if respondents had ever been diagnosed with or told they have diabetes. The case definition for minor depression required a record of ICD-9 code 311 (Depression NOS), plus a prescription for an antidepressant medication. When determining the odds of having comorbid depression, the cohort of individuals with diabetes was compared with all of the MEPS survey respondents for the entire year.

### Definition of Health Care Utilization and Expenditures

Four categories of health care utilization and expenditures were examined: 1) ambulatory care (including visits with a medical provider visits in an office or outpatient setting), 2) inpatient care (number of hospital admissions including zero-day stays), 3) emergency department visits, and 4) prescription medications received. Expenditures in MEPS are defined as the sum of payments for care, not charges or resource costs. Expenditure data include out-of-pocket payments, third-party payer payments (including Medicare, Medicaid and private insurance) and amounts for services rendered by public providers (including Department of Veterans Affairs Medical Centers). Alternative care services and over-the-counter medications are not included in MEPS total expenditures. In cases where expenditure data are missing or where care is provided under a capitated reimbursement arrangement, MEPS uses an imputation process to estimate expenditures. The process, known as the "hot-deck" method, involves matching the medical event for which expenditure data are missing with an event that has similar characteristics.[14] The amount of expenditures for the similar event is then assigned to the missing data.

### Data Analysis

Population characteristics were examined for differences between individuals with diabetes with and without depression using chi-square or t-tests as appropriate. Characteristics included age, sex, race/ethnicity, level of education, health insurance status, number of comorbidities (based on number of unique ICD-9 codes[15]), poverty level, physical and mental health status, and diabetes severity. Physical and mental health status were based on the Short-Form 12 survey's Physical and Mental Component Summary scores (SF-12 PCS and SF-12 MCS).[16] Proxy measures for diabetes severity were the presence of coronary heart disease (CHD), diabetes-related eye problems, or diabetes-related kidney problems, or use of insulin.

The prevalence of having depression in individuals with diabetes versus the general population was determined using unadjusted chi-square analysis as well as analyses adjusting for age, sex, race/ethnicity and marital status. Mean, unadjusted annual utilization rates and expenditures for ambulatory care visits, inpatient admissions, emergency department visits, and prescription medications were estimated in univariate analyses. Due to their skewed nature, expenditure data were log transformed prior to testing for statistical significance.

Multivariate regression methods for survey data were used to identify those factors that were significantly associated with resource use and log-transformed expenditures in each category. All expenditure data were expressed in 2003 US$. Resource utilization data are discrete data in that an individual can have one or two office visits, but not 1.5. In addition, many respondents have a low number of visits (e.g., zero or one), while a few people have many visits. Due to this over-dispersion of data and the fact that the data are discrete, negative binomial regression methods were used to evaluate predictors of health care utilization. Linear regression methods were used to evaluate predictors of log-transformed expenditures. For both utilization and expenditures, the predictor variable of interest was the presence or absence of diagnosed minor depression. Covariates in both models included age, sex, years of education, race/ethnicity, income level, insurance status, number of comorbid conditions, presence of diabetes complications, use of insulin, having a usual source of care, and physical and mental health status were included in the models. STATA Statistical Software was used in order to account for the MEPS survey weights and to provide nationally representative estimates. All differences between estimates were considered statistically significant at the p = 0.05 level. The Colorado Multiple Institutional Review Board approved this study.

## Results

A total of 1932 US adults with diabetes were identified, 435 with minor depression and 1497 without minor depression. Individuals with diabetes were significantly more likely to have depression than were people without diabetes (OR 2.47, 95% CI 2.17, 2.81). After adjusting for age, sex, race/ethnicity and marital status, individuals with diabetes remained almost twice as likely as individuals without diabetes to have depression (OR 1.81, C.I. 1.56–2.09).

A higher proportion of women than men were depressed (65% vs. 35%, p < 0.05). Compared to adults without depression, those with depression had lower incomes, more comorbid conditions (10.2 vs. 6.5, p < 0.05), and were more likely to use insulin (18.7% vs. 7.3%, p < 0.01). Depressed individuals were also more likely to have diabetes-related complications than those without depression (Table 1). Physical and mental health status was also higher for adults without depression compared to those with depression (41.1 vs. 35.4, p < 0.01 and 50.4 vs. 40.6, p < 0.05, respectively).

**Table 1:** Demographic and clinical characteristics of Adults with diabetes with and without comorbid depression. (United States, 2003)

Variable	Depression + n = 435	Depression - n = 1497
	
Age (mean, SE)	59.0 (0.35)	61.0 (0.62)
		
Sex (% male, SE)	35 (0.02)	51 (0.02)*
		
Education (mean years, SE)	11.6 (0.13)	11.8 (0.11)
		
Race/Ethnicity (%, SE)		
Hispanic	11 (0.04)	12 (0.04)
Black, not Hispanic	13 (0.01)	16 (0.01)
Asian, not Hispanic	1 (0.02)	5 (0.02)
White and other	75 (0.02)	67 (0.01)
		
Income level (%, SE)		
Poor (< 100% federal poverty level (fpl))	20 (0.03)	12 (0.03)*
Near poor (100–124% fpl)	7 (0.04)	6 (0.04)*
Low income (125–199% fpl)	17 (0.02)	17 (0.02)*
Middle income (200–399% fpl)	33 (0.02)	30 (0.02)*
High income (> 400% fpl)	23 (0.01)	35 (0.01)*
		
Insurance coverage (%, SE)		
Private only	59 (0.01)	64 (0.01)
Public only	36 (0.01)	29 (0.01)
None	5 (0.04)	7 (0.04)
		
Having a Usual Source of Care (%, SE)	96 (0.04)	95 (0.03)
		
# of Comorbid conditions (mean, SE)	10.2 (0.43)	6.5 (0.07)*
		
Diabetes complications (% yes, SE)		
CHD	4.0 (0.01)	12.1 (0.01)*
Eye	7.5 (0.02)	18.6 (0.02)^†^
Renal	11.3 (0.04)	20.0 (0.04)^†^
		
Insulin use (% yes, SE)	7.3 (0.03)	18.7 (0.03)^†^
		
Health Status		
SF-12 PCS (mean, SE)	35.4 (0.42)	41.1 (0.25)^†^
SF-12 MCS (mean, SE)	40.6 (1.04)	50.4 (0.17)*

* = p < 0.05; ^† ^= p < 0.01

Mean, unadjusted annual health care utilization rates and expenditures for 2003 are presented in Table 2. Depressed individuals had significantly more ambulatory care visits (17.9 vs. 11.4, p = 0.04) and more prescriptions (60.7 vs. 37.8, p = 0.05) than non-depressed individuals. Mean number of emergency department visits were higher for those with depression, with borderline significance (0.51 vs 0.31, p = 0.053). Differences between utilization rates did not reach statistical significance in any other category of care.

**Table 2:** Mean unadjusted annual health care utilization and expenditures among US adults with diabetes with and without minor depression

	Utilization*		**Expenditures**^†^	
Category	DEP +	DEP -	DEP +	DEP -
Ambulatory Care	17.9	11.4^‡^	$2,297	$1,420
Hospitalizations	0.53	0.28	$4,390	$2,684
Emergency Department Visits	0.51	0.31	$294	$166
Prescriptions	60.7	37.8^‡^	$4,061	$2,374
Total	n/a	n/a	$13,038	$8,065

* Mean units of use

^† ^Expressed as 2003 US$

^‡ ^= p ≤ 0.05

Multivariate regression results identifying factors associated with resource use and log-transformed expenditures are shown in Tables 3 and 4, respectively. In adjusted analyses, comorbid minor depression was not significantly associated with resource utilization or expenditures in any category, including total expenditures (data not shown). Factors significantly associated with utilization in adjusted regressions were education (ambulatory care), race/ethnicity (prescriptions), income (prescriptions), having a usual source of care (hospitalizations), comorbidities (all four categories), CHD diagnosis (prescriptions), insulin use (hospitalizations and prescriptions), physical health status (emergency visits and prescriptions), and mental health status (hospitalizations, emergency visits, and prescriptions). Factors significantly associated with expenditures in fully adjusted models were age (emergency visits and prescriptions), sex and education (ambulatory care), race/ethnicity (prescriptions), income (ambulatory care), insurance (emergency visits and hospitalizations), number of comorbidities (ambulatory care and prescriptions), diabetes-related eye problems (emergency visits and hospitalizations), insulin use (prescriptions), and physical health status (prescriptions). Insurance status, number of comorbidities, physical health status, and insulin use were significantly associated with higher total expenditures (data not shown).

**Table 3:** Factors associated with health care utilization by category by US adults with diabetes, 2003

	Ambulatory Care		Hospitalizations		Emergency Visits		Prescriptions	
Variable	β Coeff	95% CI	β Coeff	95% CI	β Coeff	95% CI	β Coeff	95% CI
	
Depression	-0.02	-0.17, 0.12	-0.19	-0.73, 0.34	-0.014	-1.18, 0.90	0.09	-0.16, 0.34
								
Age	0.002	-0.005, 0.01	0.02	-0.01, 0.04	-0.002	-0.02, 0.02	0.003	-0.001, 0.01
								
Sex	0.05	-0.10, 0.19	-0.15	-0.94, 0.63	0.11	-0.24, 0.46	0.01	-0.02, 0.04
								
Years of Education	0.04	0.03, 0.04 ^†^	-0.04	-0.12, -0.05	-0.02	-0.14, 0.11	-0.002	-0.03, 0.02
								
Race/Ethnicity								
Black, not Hispanic	-0.09	-0.36, 0.17	0.26	-0.46, 0.98	0.20	-0.37, 0.77	0.27	-0.06, 0.60
Asian, not Hispanic	-0.01	-0.017, 0.14	-0.09	-1.13, 0.96	-0.06	-1.61, 1.48	0.15	-0.07, 0.36
White/Other, not Hispanic	-0.01	-0.020, 0.18	0.23	-1.20, 1.65	0.05	-0.86, 0.95	0.25	0.02, 0.48*
								
Income level								
Near poor	-0.33	-0.73, 0.07	-0.18	-1.85, 1.49	-0.46	-2.40, 1.49	-0.37	-0.61, -0.14*
Low Income	-0.10	-0.53, 0.33	-0.11	-0.34, 0.12	0.002	-0.35, 0.36	-0.11	-0.63, 0.40
Middle Income	-0.09	-0.51, 0.33	-0.18	-1.14, 0.77	-0.27	-1.24, 0.70	-0.09	-0.30, 0.11
High Income	-0.014	-0.59, 0.31	-0.38	-1.39, 0.64	-0.50	-1.67, 0.68	-0.14	-0.26, -0.01*
								
Insurance status								
Public only	-0.14	-0.54, 0.25	-0.20	-0.42, -0.01	-0.10	-1.47, 1.26	0.14	-0.12, 0.40
No insurance	-0.38	-1.71, 0.96	-0.37	-1.75, 1.00	-0.24	-1.51, 1.02	-0.14	-0.51, 0.23
								
Have usual source of care	-0.22	-1.44, 1.00	0.74	0.59, 0.90^†^	0.31	-0.87, 1.49	-0.44	-0.81, -0.06
								
# of Comorbid conditions	0.08	0.05, 0.12 ^†^	0.12	0.09, 0.15^†^	0.08	0.04, 0.11*	0.06	0.04, 0.08^†^
								
DM complications								
CHD	0.04	-0.18, 0.27	-0.02	-0.15, 0.12	0.16	-0.18, 0.50	-0.12	-0.21, -0.03*
Eye	0.06	-0.24, 0.36	0.05	-0.30, 0.41	-0.08	-0.39, 0.22	-0.05	-0.11, 0.01
Renal	0.00	-0.83, 0.84	-0.10	-1.17, 0.96	-0.03	-0.53, 0.47	-0.01	-0.10, 0.07
								
Insulin use	0.07	-0.04, 0.17	-0.40	-0.68, -0.11*	-0.33	-0.95, 0.28	-0.16	-0.28, -0.05*
								
SF-12 PCS	-0.01	-0.02, 0.008	-0.01	-0.04, 0.01	-0.01	-0.03, -0.0003*	-0.01	-0.01, -0.005^†^
SF-12 MCS	-0.004	-0.01, 0.004	-0.019	-0.023, -0.016^†^	-0.01	-0.03, -0.001*	-0.001	-0.002, 0.001*

* = p < 0.05; ^† ^= p < 0.01; ^‡ ^= p < 0.001

Reference groups: no depression, female, race/ethnicity = Hispanic, income level = poor, private insurance, no usual source of care, no CHD, no eye disease, no renal disease, no insulin use Abbreviations: DM = diabetes mellitus; SF-12 PCS = Short From 12 Physical Component Summary; SF-12 MCS = SF-12 Mental Component Summary; CHD = coronary heart disease

**Table 4:** Factors associated with health care expenditures by adults with diabetes in 2003.*

	Ambulatory Care		Hospitalizations		Emergency Visits		Prescriptions	
Variable	β Coeff	95% CI	β Coeff	95% CI	β Coeff	95% CI	β Coeff	95% CI
Depression	0.01	-0.06, 0.05	-0.02	-0.15, 0.10	0.04	-0.48, 0.57	0.10	-0.02, 0.23
								
Age	0.002	-0.005, 0.01	0.01	-0.02, 0.03	-0.01	-0.01, -0.003^†^	0.002	0.000, 0.005^†^
								
Sex	-0.03	-0.06, 0.01^†^	-0.10	-0.33, 0.12	-0.11	-0.44, 0.23	-0.05	-0.15, 0.06
								
Years of Education	0.02	0.00, 0.05^†^	0.005	-0.01, 0.02	-0.02	-0.06, 0.01	0.002	-0.01, 0.02
								
Race/Ethnicity								
Black, not Hispanic	-0.07	-0.18, 0.04	0.27	-0.20, 0.73	-0.01	-0.24, 0.22	0.13	-0.01, 0.28
Asian, not Hispanic	0.01	-0.41, 0.42	-0.09	-1.27, 1.09	0.45	-0.48, 1.38	0.12	-0.23, 0.47
White/Other, not Hispanic	0.001	-0.25, 0.25	0.04	-0.30, 0.39	0.04	-0.20, 0.28	0.15	0.06, 0.24^†^
								
Income level								
Near poor	-0.11	-0.22, 0.004	0.20	-0.19, 0.59	0.08	-0.20, 0.35	-0.13	-0.33, 0.07
Low Income	-0.11	-0.20, -0.01^†^	0.11	-0.06, 0.29	0.21	-0.52, 0.94	-0.07	-0.21, 0.07
Middle Income	0.02	-0.09, 0.13	0.04	-0.09, 1.70	0.11	-0.27, 0.49	-0.04	-0.26, 0.18
High Income	-0.06	-0.12, 0.00^†^	-0.05	-0.36, 0.27	0.08	-0.57, 0.72	-0.06	-0.19, 0.08
								
Insurance status								
public only	-0.09	-0.25, 0.07	-0.15	-0.22, -0.08^†^	-0.14	-0.28, -0.003^†^	0.06	-0.20, 0.32
no insurance	-0.26	-0.78, 0.26	-0.56	-0.96, -0.14^†^	-0.36	-0.51, -0.20^‡^	-0.06	-0.43, 0.30
								
Have usual source of care	-0.08	-0.81, 0.66	-0.22	-0.96, 0.52	0.35	-0.40, 1.10	-0.11	-0.43, 0.22
								
# of Comorbid conditions	0.06	0.05, 0.06^‡^	0.01	-0.01, 0.03	0.01	-0.04, 0.05	0.04	0.03, 0.04^§^
								
DM complications								
CHD	-0.02	-0.16, 0.12	-0.03	-0.09, 0.03	0.06	-0.53, 0.65	-0.06	-0.15, 0.04
Eye	0.02	-0.01, 0.05	0.11	0.01, 0.21^†^	-0.13	-0.17, -0.08^‡^	-0.04	-0.16, 0.19
Renal	-0.04	-0.42, 0.34	-0.08	-0.19, 0.02	-0.06	-0.71, 0.58	0.004	-0.12, 0.13
								
Insulin use	-0.01	-0.10, 0.07	-0.19	-0.54, 0.15	0.09	-0.13, 0.30	-0.13	-0.19, -0.07^†^
								
SF-12 PCS	-0.002	-0.01, 0.01	0.0007	-0.002, 0.003	-0.003	-0.01, 0.001	-0.004	-0.01, -0.0001^†^
SF-12 MCS	-0.002	-0.01, 0.004	0.002	-0.01, 0.01	0.001	-0.01, 0.01	0.0002	-0.004, 0.004

* Expenditures log transformed to account for skewed data

^† ^= p < 0.05; ^‡ ^= p < 0.01; ^§ ^= p < 0.001

Reference groups: no depression, female, race/ethnicity = Hispanic, income level = poor, private insurance, no usual source of care, no CHD, no Eye disease, no renal disease, no insulin use Abbreviations: ; SF-12 PCS = Short From 12 Physical Component Summary; SF-12 MCS: SF-12 Mental Component Summary; CHD = coronary heart disease

## Discussion

US adults with diabetes were nearly twice as likely to have minor depression compared with the general population in 2003. These results are consistent with previous reports of increased depression prevalence among those with diabetes. In a meta-analysis of 42 eligible studies, 18 included a non-diabetic control group and provided data sufficient to calculate a pooled odds ratio.[8] Results of that study indicated that among people with diabetes, the odds of having depression were twice that of those without diabetes. The finding in the current study of an increased prevalence of minor depression among people with diabetes in a nationally representative database reinforces the need to consider comorbid depression when treating patients with diabetes for a number of reasons. First, comorbid depression in people with diabetes has been associated with poor glycemic control. Lustman and colleagues report, also based on a meta-analysis, that adults with diabetes and depression were more likely to have elevated glycosylated hemoglobin (HbA1c) than those without depression.[10] Results of increased HbA1c for diabetes with compared to without depression were consistent for both type1 and 2 diabetes, and for depression assessed by self-report and diagnosis codes. A number of studies suggest that the mechanism for poorer glycemic control among those with diabetes with versus without depression may be related to the impact of depression on self-care activities. Depression and diabetes is associated with poorer adherence to medication, diet, and exercise regimens.[9,17,18]

Secondly, an association between comorbid depression and diabetes and increased mortality has been proposed in a number of studies. Katon, et al, reported that younger age is associated with major depression among people with diabetes receiving care through a managed care organization in Washington state.[19] Similar results have been reported for patients with minor depression and diabetes using data from the Medical Expenditure Panel Survey.[20] Finally, Zhang demonstrated an increased risk for mortality among people with versus without major depressive symptoms based on analyses using the National Health and Nutrition Examination Survey.[21] Together, these results present a compelling need to screen for, diagnose, and effectively treat depression among people with diabetes. Screening for depressive symptoms could be incorporated into patient care activities with screening tools. One such instrument is the PHQ-2, a validated 2 question screen that can identify patients with diabetes that may benefit from further evaluation for comorbid depression. [22-24]

In adjusted analyses, no significant associations exist between comorbid depression and total expenditures, expenditures in any category, or resource utilization in any category. An increased number of comorbid conditions were associated with increased utilization in all four categories. Health status was associated with hospitalizations, emergency visits, and prescription use. Higher levels of physical functioning were associated with decreased use of emergency visits and prescriptions, while higher mental functioning was associated with decreased use in all categories except ambulatory care visits. Increased prescription use was associated with being white (not Hispanic) compared to Hispanic ethnicity. Being near poor or having high income were both associated with decreased use of prescription medications compared to the lowest income category. Because use of prescription medications requires access to physician services, the potential exists that those in the near poor category have difficulty either seeing a prescribing health provider and/or affording the out-of-pocket costs of medications. Those in the highest income category may be in overall better health. These issues warrant further study.

In adjusted analyses evaluating factors associated with expenditures, an increased number of comorbid conditions was associated with increased expenditures for ambulatory visits and prescription medications. Having public only or no insurance was associated with lower expenditures for inpatient and emergency care. No other consistent associations were observed between covariates and expenditures in more than one category.

These results differ from those previously reported from the 1996 MEPS data,[6] perhaps due to the fact that our analyses adjusted for more covariates. The earlier reported analyses adjusted for age, sex, race/ethnicity, health insurance, and number (ranging from one to seven) of comorbid chronic conditions.[6] Multivariate regressions in the current study adjusted for additional variables including education, income, the presence of diabetes-related complications, and physical and mental health status. Variables relating to diabetes-related complications and health status were not available in the 1996 MEPS database. Factors that were significant predictors of total costs for US adults with diabetes in 2003 were insurance status, number of comorbidities, physical health status, and insulin use.

US adults with diabetes are twice as likely to have comorbid minor depression as US adults without diabetes. As discussed earlier, comorbid depression has a deleterious impact on adherence to medication and medical regimens, and is associated with poor glycemic control. Ongoing research is necessary to elucidate both the causal relationship between diabetes and depression, as well as identify interventions to treat depression effectively in adults with diabetes. The American Diabetes Association recommends that all factors, clinical as well as psychosocial, should be considered when developing treatment plans for people with diabetes.[25] Screening for and treating depression in the diabetes population is an important component of care for the whole patient.

Another area warranting additional research is the potential that the association between depression and glycemic control is mediated through the deleterious impact of depression on self-care behaviors. As discussed earlier, depression among people with diabetes is associated with poor self-care behaviors. Improving self-care behaviors has been shown to improve glycemic control, supporting the need for ongoing research to confirm the links between depression, diabetes self-management, and glycemic control, as well as research to investigate whether interventions treating depression can improve self-care and glycemic control.

In addition to providing a basis for future research, there are several strengths of this study. The MEPS database is a large, nationally representative sample of the United States civilian non-institutionalized population, with an over-sampling of black and Hispanic subpopulations. The results are generalizable and the study was able to incorporate appropriate adjustments for demographic characteristics, such as age, sex, and race/ethnicity, as well as clinical characteristics, such as number of comorbidities.

The potential exists for underreporting of either diabetes or depression in the MEPS database, either of which would have limited the sample size of this study. A diagnosis of depression is particularly vulnerable to this limitation due to under-diagnosing and potential stigma that may be attached to mental disorders. The case definition for minor depression used in the current study required a diagnosis code and use of a prescription antidepressant, cases included here are likely to be accurately assessed as being depressed. A potential limitation of this restrictive case definition is that individuals with undiagnosed depression, as well as those with a diagnosis but without treatment with prescription medication will be misclassified as having diabetes without depression. The 2003 MEPS database does not include data obtained by screening respondents for depressive symptoms, which may be a limitation of these analyses. However, we report prevalence estimates for comorbid depression consistent with published studies, despite the potential for misclassification. A further source of misclassification in this study is the inclusion of adults with diabetes with major depression in the non-depressed (i.e., depression -) group. Based on a post-hoc analysis, only 19 individuals had diagnosis codes for diabetes and major depression in the 2003 MEPS database. Therefore, the impact of any misclassification of respondents with major depression is likely to be minimal. Finally, MEPS includes only 3-digit ICD-9 codes, preventing a classification of Type 1 or Type 2 diabetes. Due to the relatively low prevalence of Type 1 diabetes (5–10% of all people with diabetes), the impact of this limitation is expected to be minimal.[1]

## Conclusion

People with diabetes are twice as likely to be depressed as the general population, a factor to be considered when developing diabetes treatment programs. Screening for and treatment of depression is appropriate, as is additional research into a causal relationship between diabetes and depression.

## Competing interests

The author(s) declare that they have no competing interests.

## Authors' contributions

LN participated in study conduct and design, results interpretation, manuscript preparation and revisions. PLB participated in study design, results interpretation, manuscript review and revisions. JG participated in study design, results interpretation, manuscript review and revisions. MM conceived of the study and was responsible for oversight of study design and conduct, interpreting the results, and manuscript revisions. All authors read and approved the final manuscript.
